# Impact of Covid-19 on attendances for a 1^st^ episode of reduced fetal movements: A retrospective observational study

**DOI:** 10.1371/journal.pone.0253796

**Published:** 2021-06-25

**Authors:** Laia Marques-Fernandez, Swati Sharma, Una Mannu, Hsu Phern Chong

**Affiliations:** Department of Obstetrics and Gynaecology, Addenbrooke’s Hospital, Cambridge University Hospitals NHS Foundation Trust, Cambridge, Cambridgeshire, United Kingdom; Kobe University Graduate School of Medicine School of Medicine, JAPAN

## Abstract

**Background:**

Prior studies have demonstrated an increased stillbirth rate. It was suggested that the COVID-19 pandemic may have impacted on attendances for reduced fetal movements. Thus, we sought to ascertain the impact of the pandemic on attendances for reduced fetal movements (RFM) in our unit, ultrasound provision for reduced fetal movements, and the stillbirth rate.

**Methods:**

This was a single site retrospective cohort study involving all women complaining of a 1^st^ episode of reduced fetal movements between 01/03/2020-30/04/2020 (COVID) to 01/03/2019-30/04/2019 (Pre-COVID). Data were retrieved from computerised hospital records and statistical analyses were performed using GraphPad Prism and SPSS.

**Results:**

22% (179/810) of women presented with a 1^st^ episode of reduced fetal movements Pre-COVID compared to 18% (145/803) during COVID (p = 0.047). Primiparous women were significantly over-represented in this population with a 1.4-fold increase in attendances during COVID (67% vs 48%, p = 0.0005). Neither the total stillbirth rate nor the stillbirth rate amongst women who presented with reduced fetal movements changed during COVID. Ultrasound provision was not impacted by COVID with 95% of the scans performed according to local guidelines, compared to Pre-COVID (74%, p = 0.0001).

**Conclusions:**

There is a significant decrease in 1^st^ attendances for reduced fetal movements during COVID-19 pandemic. Primiparous women were 1.4 times more likely to attend with RFM. Women should be reassured that COVID-19 has not resulted in a decreased provision of care for RFM, and has not impacted on the stillbirth rate.

## Introduction

The first reported case of a novel coronavirus infection was reported at the end of 2019 and occurred in Wuhan, China. The virus responsible for the disease was a new strain of coronavirus, since named as severe acute respiratory syndrome coronavirus 2 (SARS-CoV-2). On the 11th of March 2020, the World Health Organisation (WHO) declared a pandemic [1]. On the 23rd of March 2020, the UK government declared a lockdown and outdoor excursions were limited to those strictly necessary [2]. Health care provision changed dramatically in both primary and secondary care. Antenatal services were no different and most of the clinic appointments were moved to a telephone based service or video-conferencing.

The unintended effects of the lockdown on pregnancy outcomes are unknown. Certainly in other specialties, delayed presentation for a variety of conditions has been reported [3, 4]. The number of people attending emergency departments in England with symptoms of a possible heart attack halved following the lockdown [5]. Staff illness too, impacted upon provision of services [6]. At a tertiary maternity unit in London, Khalil et al reported an increase in the stillbirth rate when comparing rates immediately before and during the lockdown [7]. Concerns were raised about whether women were fearful of accessing maternity services during the lockdown.

Fetal movements are considered as a sign of fetal well-being [8, 9], although, RFM is not the causative of still-birth [10]. 55% of women who had stillbirth perceived RFM prior to diagnosis [11]. In line with RCOG guidelines, women with reduced fetal movements after 28+0 weeks of gestation—when fetal movements should have acquired a regular pattern—are advised to attend their maternity unit for a fetal assessment. This includes a handheld Doppler, a cardiotocograph (CTG), and if indicated, an ultrasound assessment (USS) [12]. The CTG can only be interpreted by senior clinicians in specialised units. That is why, to the best of our knowledge, all RFM assessments in our community are referred to our centre. Our hospital is the only unit equipped to assess RFM in the surrounding area, hence we chose RFM as a measure of the quality of provision of maternity services.

## Materials and methods

This work was registered as service evaluation project. All women booked for maternity care at the Rosie Maternity Hospital are asked verbally whether they give their consent for data to be used for research and this is recorded in their computerised hospital records. Identifiable data were removed from cases to ensure anonymity. Additionally, in accordance with the United Kingdom National Health Service National Research Ethics Service guidance, neither individual informed consent nor formal research ethics committee review was required, because the study was undertaken by the direct clinical team using information collected in the course of routine care.

The Rosie Maternity Hospital (part of Cambridge University Hospital NHS Foundation trust, England) is a tertiary care hospital with an annual delivery rate of 5200 births. It serves a population of 6.23 million in the East of England and runs a 24 hours service for reduced fetal movements.

In our unit, the criteria for USS following a first episode of RFM include: primiparity, women with one or more identified risk factors for stillbirths, ongoing maternal concerns with RFM in the presence of a normal CTG in a multiparous woman. Women who present at gestational ages of ≥ 38+6 induction of labour may be offered instead. We defined risk factors for still-birth as primiparity, previous caesarean section (CS), body mass index (BMI) ≥ 35, Afro-Caribbean ethnicity, smoking during pregnancy, social factors such as poor attendance with antenatal care (ANC), previous stillbirth, diabetes (GDM, Pre-existing), hypertension (pre-existing hypertension, pre-eclampsia, pregnancy induced hypertension), and continued absence of fetal movements. In our unit, USS should be performed within 3 days of the first episode of RFM, and this is in line with practices elsewhere [13]. The indications for USS or the advice given to pregnant women did not differ across periods.

We were unable to assess the proportion of women who had RFM but chose not to attend as it is not possible to capture this information. We also evaluated the total number of stillbirth that occurred in the periods specified. We chose 01/03/2020-30/04/2020 to include women in the run up to the UK wide lockdown which was introduced on 23/3/2020, and in the ensuing 5 weeks. Furthermore, the two other studies available on the subject whilst we were designing our study included time frames that started from January 2020 [14] and February 2020 [7], respectively. Therefore, we chose a similar period. Moreover, our aim was not to assess the influence of lockdown, but the influence of COVID19 itself in the provision of care. COVID19 already had a direct impact on our daily lives and practice before the instauration of lockdown [15]. Some countries were already in lockdown and the virus was in the community since late January [16], hence the perceived fear already kept individuals from attending the hospital. We selected a similar epoch in 2019 as the control, due to possible seasonal variations in attendances for RFM, and pregnancy outcome (01/03/2019–30/04/2019). Our aim in doing so was to limit the confounding factors in order to assess the impact of the pandemic itself in the attendances. We hypothesised that fewer patients attended our unit during the COVID period due to the fear of COVID19 exposure and to the concern of putting the NHS under more strain.

We used the 1st episode of RFM as a reference point, which triggers the aforementioned management and investigations. This can vary in subsequent episodes. We then analysed our compliance against the guidelines from the 1st episode until birth and its relationship with birth outcomes. We excluded women who presented with their 2^nd^ or 3^rd^ episode of RFM, had fetus(es) with a known congenital anomaly, presented under 28 completed weeks gestation, and who attended in labour and also complained of RFM.

The required size of our study was calculated aiming for a power of 0.80. We used an online tool hosted by the University of Vienna [17] that calculates the minimum sample size for studies using Fisher’s exact test (the statistical method we applied). According to which, the total sample size required for our study should be more than 1000 participants and our total sample size is 1603. This number is equivalent to the sum of the patients that delivered in our unit from 1/3/19 to 30/4/19 and from 1/3/20 to 30/4/20 (as mentioned in the “Results” section). Within each cohort, we then analysed the data of patients that presented with their first episode of RFM.

Based on an adjusted stillbirth rate of 3.26/1000 in the Cambridgeshire region [18] we would expect our work to be sufficiently powered to detect a change in the stillbirth rate between the two epochs studied.

Data were extracted from computerised hospital records for patient demographics, risk factors for stillbirth, results from USS, time interval between the first attendance for RFM and USS, further management of pregnancy and its outcome, gestational age and birthweight at delivery, and finally admission to the neonatal unit. Data were tabulated using Microsoft Excel. Continuous variables are presented as mean and standard deviation for parametric data, and the significance of the results was determined using Fisher’s exact test. Non-parametric data are presented as median and interquartile range, and the significance of the results was determined using Mann-Whitney U test. Percentages were rounded up or down to the nearest absolute number for data consistency where appropriate. IBM SPSS Statistics for Macintosh, version 26 (IBM Corp., Armonk, N.Y., USA) was used for statistical analysis. GraphPad Prism (version 5, San Francisco, La Jolla California USA) was used to replicate the results by another author.

## Results

Similar numbers of women delivered during the Pre-COVID (n = 810) and COVID periods (n = 803). A total of 179 and 145 women presented with their 1^st^ episode of RFM ≥ 28 weeks gestation in the Pre-COVID and COVID periods respectively. Using the proportion of women who delivered during the periods specified as the denominator, this translates to a 4% reduction in the incidence of a first presentation of RFM from 22 to 18% respectively (p = 0.047). Repeat attendances were similar with 32% (46/145) of women reattending during COVID compared with Pre-COVID [30% (54/179, p = 0.81)].

However, there was a higher proportion of primiparous women presenting with RFM during the COVID period 67% (97/145) than in the Pre-COVID period 48% (85/179, p = 0.0005). Patient demographics are presented in Table 1 below. There were no significant differences in the median maternal age, nor the gestational age at the time of presentation between the two cohorts. One woman in each epoch had a multiple pregnancy and both were dichorionic diamniotic (DCDA). There were no significant differences in risk factors for stillbirth between cohorts.

**Table 1:** Patient characteristics.

	2019 (Pre-COVID19) N = 179 (%)	2020 (COVID19) N = 145 (%)	p value
**Age**	31 (27–35)	31 (27–34)	0.395^a^
**Gestational age**	36 (31–38)	35 (31–38)	0.934^a^
**Ethnicity**			
**White British**	126 (70)	91 (63)	0.156
**Other white background**	26 (15)	30 (21)	0.183
**Other**	27 (15)	24 (17)	0.292
**Asian background**	17	20	
**Black background**	5	1	
**Other ethnic group**	3	2	
**Mixed background**	2	1	
**Parity**			
**Primiparous**	85 (48)	97 (67)	**0.0005**
**Multiparous**	94 (53)	48 (33)	
**Risk factors for stillbirth**			
**Smoking during pregnancy**	10 (6)	13 (9)	0.28
**BMI ≥ 35**	19 (11)	12 (8)	0.570
**Diabetes**	10 (6)	12 (8)	0.379
**GDM**	9 (5)	7 (5)	
**Type 1**	1 (1)	2 (1)	
**Type 2**	0 (0)	1 (1)	
**Hypertension at time of presentation for RFM**	4 (2)	6 (4)	0.352
**PIH**	4 (2)	3 (2)	
**Essential**	0 (0.0)	2 (1)	
**Hypertension PET**	0 (0.0)	1 (1)	
**Previous IUD**	0 (0.0)	2 (1)	0.200
**Recurrent miscarriages**	3 (2)*	4 (3)	0.705

*Table 1*: *Demographics*. *Data are reported as median (Inter-Quartile-Range (IQR = Q1-Q3)) or as N (%)*, *as applicable*. *p-Values are from Fisher’s exact tests*, *unless stated otherwise*. *Bold p-values are significant at p < 0*.*05*. *(a) Mann-Whitney U test*. *Pre-COVID19 covered a time span of 8 weeks from 01/03/2019-30/04/2019*. *COVID19 covered a time span of 8 weeks from 01/03/2020-30/04/2020*.

The total number of stillbirths in the Pre-COVID period was 6, and 4 during COVID. The overall incidence of stillbirth therefore, was 7 per 1000 livebirths Pre-COVID, and 4 per 1000 during COVID. Of these, 50% (3/6) of had attended with a 1^st^ presentation of RFM in the Pre-COVID period, and 25% in the COVID period (1/4).

### Interval to ultrasound scan assessment

The percentage of women having an USS assessment for RFM Pre-COVID was 112/179 (63%) and this was unchanged during COVID 63% (92/145, p = 1.00) (Table 2). However, during COVID, 95% (87/92) of women had an USS within 3 days, representing a 21% increase compared to Pre-COVID [74% (83/112, p = 0.0001)]. 32% (28/87) had an USS within 24 hours during COVID and 22% (19/83) Pre-COVID. Our guidelines state that indicated USS should be done within 3 working days from presentation of RFM. However, 29/112 (26%) pre-COVID and 5/92 (5%) during COVID (p = 0.0001) had an USS more than 3 days after presentation. The reasons for the delay are both logistical (e.g. workload) and related to human error (e.g. inaccurate USS request).

**Table 2:** USS findings following a 1^st^ presentation of reduced fetal movements.

	2019 (Pre-COVID19)	2020 (COVID19) N = 92 (%)	p value
	N = 112 (%)		
**Interval between presentation and USS (days)**			
**≤ 3 days**	83 (74)	87 (95)	**0.0001**
**> 3 days**	29 (26)	5 (5)	
**USS characteristics**			
**Normal USS after the 1**^**st**^** episode of RFM**	108 (96)	86 (93)	0.91
**Any one USS feature abnormal**	4 (4)	6 (7)	0.22
**SGA**	1 (1)	1 (1)	
**Umbilical PI ≥ 97**^**th**^** centile**	0 (0)	1 (1)	
**AFI ≤ 5**	0 (0)	0 (0)	
**AFI >25**	3 (2.7)	4 (4.3)	

*Table 2*: *Ultrasound data*. *Data are reported as median (IQR) or as N (%)*, *as applicable*. *p-Values are from Fisher’s exact tests*, *unless stated otherwise*. *Bold p-values are significant at p < 0*.*05*. *USS characteristic not assessed where figures do not add up to the total N (a) Mann-Whitney U test*.

* A normal USS was defined as having all the following parameters within the normal range: *estimated fetal weight (EFW)*, *liquor volume (AFI 6–25) and umbilical artery pulsatility index*. *Pre-COVID19 covered a time span of 8 weeks from 01/03/2019-30/04/2019*. *COVID19 covered a time span of 8 weeks from 01/03/2020-30/04/2020*.

### Pregnancy outcomes

Pregnancy outcome data were available for 98% (175/179) of women from the Pre-COVID cohort and 99% (143/145) from the COVID cohort respectively. The remaining six women delivered elsewhere and therefore pregnancy outcomes were not available (Table 3).

**Table 3:** Pregnancy and neonatal outcomes.

	2019 (Pre-COVID19)	2020 (COVID19) N = 145 (%)	p value
	N = 179 (%)		
**Mode of delivery**			
**Total induction of labour**	97 (54)	72 (49)	0.372
**RFM alone**	41	37	
**RFM with another medical indication**	11	8	
**Not due to RFM**	45	27	
**Spontaneous vaginal deliveries**	96 (54)	83 (57)	0.574
**Operative vaginal delivery**	32 (18)	20 (14)	0.363
**Vacuum**	12 (7)	6 (4)	
**Forceps**	20 (11)	14 (10)	
**Low-segment C-section**	47 (26)	40 (28)	0.802
**Lost to follow up**^**a**^	4 (2)	2 (1)	0.695
**EBL (mL)**	400.0 (250–600)	400.0 (250–700)	0.276^b^
**GA at birth (weeks)**	40.0 (38–41)	40.0 (38–40)	0.917^b^
**Birth weight (g)**	3470 (3128–3811)	3405 (3089–3676)	0.205^a^
Neonatal morbidity*			
**None**	151 (84)	129 (88)	0.256
**Admission to NICU**	21 (12)	8 (5)	0.077
**Antenatal stillbirth**	3 (2)	1 (1)	0.631

*Table 3*: *Pregnancy and neonatal outcomes*. *Data are reported as N (%) or median (IQR)*, *as applicable*. *p-Values are from Fisher’s exact tests*, *unless stated otherwise*. *Bold p-values are significant at p < 0*.*05*.

*(a) Patients who transferred care to another hospital and were lost to follow up*.

*(b) Mann-Whitney U test*. *Pre-COVID19 covered a time span of 8 weeks from 01/03/2019-30/04/2019*. *COVID19 covered a time span of 8 weeks from 01/03/2020-30/04/2020*.

*Neonatal morbidity was calculated as a proportion of all babies delivered to women who presented with RFM during the periods studied as this included 1 pair of DCDA twins in each epoch (n = 180 Pre-COVID, *and n = 146 during COVID)*.

Induction of labour did not vary significantly between the two cohorts. In 2019, 54% (97/179) women were induced compared to 49% (72/145) in 2020 (p = 0.372). We observed similar rates of Caesarean Section, vaginal deliveries, and instrumental deliveries between the two cohorts.

### Neonatal outcomes

The median birthweight was 3470 g (IQR 3128–3811) for the Pre-COVID cohort and 3405 g (3089–3676) for the COVID cohort (p = 0.205). GA at birth was of 40.0 (IQR 38–41) and 40.0 (IQR 38–40) (p = 0.917). Neonatal admission rates did not differ significantly between the two cohorts (Table 3).

## Discussion

The purposes of our work were to identify the unintended effects of lockdown on pregnant women’s attendances for RFM as this was proposed as a possible cause for the observed increased stillbirth [14]. A possible explanation for the observed increased incidence, was the time period selected as a comparator. Every maternity unit in England has an individualised background stillbirth rate. This is collected centrally through the National Perinatal Epidemiology Unit and reported in “Mothers and Babies: Reducing Risk through Audit and Confidential Enquiry (MBRRACE)” [19]. Data are collated to provide an annualised rate per 1000 births. It is possible therefore, that the observed increase in stillbirth previously reported [7], was merely a natural fluctuation in the unit rate as opposed to a true effect of the pandemic as they had compared the stillbirth rate to the immediately preceding 19 weeks. We have observed spikes and troughs in the stillbirth rate in our unit over the last 5 years (data unpublished but available upon request). Although we have predominately assessed the impact of COVID on attendances for a 1st episode of RFM, we also evaluated the change in the stillbirth rate in the preceding 12 months prior to lockdown. Contrary to Khalil et al, we found a reduction in the total stillbirth rate and this is unlikely to be due to temporal short term fluctuations in the incidence. One of the mechanisms of reducing the stillbirth rate is to increase the rate of intervention, for example by inducing labour. We did not observe an increase in the induction of labour rate during the COVID period for the women in our cohort. Our findings mirror that of the national study in England [20] and reassuringly, no increase in the stillbirth rate was identified in our unit. We accept that RFM is not causative of stillbirth, as demonstrated by a recently published large RCT on RFM [10], and we would echo these findings.

The second other study which also showed an increased stillbirth rate during the pandemic, was conducted in Nepal, a country that has different healthcare resources compared to the UK [21]. Therefore, there may have been other challenges faced by both women and healthcare professionals during the pandemic there.

We chose to exclude women with a prior presentation of RFM as these women would already have a plan of care in place, and we would not have been able to assess the impact of COVID on USS provision. In our unit, we observed a marginal reduction in attendances for RFM, but an over-representation of primiparous women in the cohort who attended. Indeed, primiparous women were 1.4 times more likely to present with RFM compared to multiparous women during COVID. As previous work have repeatedly demonstrated no significant differences in parity with regards to attendances for RFM [22, 23], we believe our findings to be true.

The increased provision of USS within 3 working days may in part, be attributed to reduced attendances for RFM itself, thus resulting in lower demand. However, as our usual practice is to offer USS assessment to all primiparous women who attend following a 1st episode of RFM, this would have resulted in increased demand for USS instead. We are therefore able to conclude that the COVID pandemic did not impact on our provision of USS for RFM in our unit. Finally, although previous studies have demonstrated an association between RFM and USS findings such as oligohydramnios and SGA [24], our findings did not reflect this. Over 90% of our cohort from both the Pre-COVID and COVID periods had normal ultrasound scan findings.

### Strengths

The strengths of our work are four-fold. Firstly, fully computerised electronic maternity records allowed completeness of data collection. This enabled us to accurately assess outcomes for women attending with reduced movements and for women that suffered a stillbirth. In our own personal experience of working in different maternity units around England, attendances for reduced fetal movements are not routinely captured. Whereas in our current unit all telephone calls to the maternity assessment unit are logged. Thus, we were able to assess attendances for all 1^st^ episodes of reduced fetal movements, and not biased towards women who were offered USS due to RFM, or for induction of labour for RFM, for example. Secondly, although the background rate of stillbirth in Cambridgeshire is low (1 in 270 women) [19], we believe our sample size to be sufficient to detect a change in the stillbirth rate as over 800 women delivered during the study periods. Thirdly, the time epochs compared accounted for any potential seasonal variations in service provision, deliveries and pregnancy outcomes. A number of studies have reported on the possible impact of seasonality on birth outcomes such as preterm birth/span [25], birth weight [26] and stillbirth [27]. Though seasonal variation in the UK stillbirth rate has never been reported, we took the precautionary measure and selected an identical epoch from the preceding year. Finally, no other study has evaluated the impact of the Covid-19 pandemic on service provision for RFM. Thus we believe our work to be both informative and unique.

### Limitations

The localised nature of data collection means that our data should be interpreted with caution. Local units should evaluate their own data with regards to provision of care for RFM. The likelihood that services could be impacted by the second wave, and with challenges associated with testing warrants further investigation. We did not evaluate women’s experiences during COVID but are aware of research being undertaken by The Health Improvement Studies Institute [28]. This may shed light on future directions for the provision of antenatal care as the UK enters a second wave.

## Conclusions

Reassuringly, we have not identified an increased incidence of stillbirth during the COVID and Pre-COVID periods. Though attendances for a 1^st^ episode of RFM reduced during the pandemic, our data demonstrates an increasing need to support women in their 1^st^ pregnancy who represented 70% of the women who attended. Caregivers may wish to consider increasing face to face contact for this population to provide them with the necessary reassurance, both in the short and longer term.

## Supporting information

S1 Data (XLSX)
